# Unraveling the Photocatalytic Performance of La_2_O_3_ Nanoparticles for the Degradation of Six Organic Dyes

**DOI:** 10.1021/acs.langmuir.5c01655

**Published:** 2025-06-13

**Authors:** Rahul S, Amal George, Suresh Babu R, Dhayal Raj A, Jayakumar G, Adarsh Rag S

**Affiliations:** † Department of Physics, Sacred Heart College (Autonomous), Tirupattur, Affiliated to Thiruvalluvar University, Serkadu, Vellore 632115, Tamil Nadu, India; ‡ Department of Mechanical Engineering, 7235 University of Saskatchewan, Saskatoon S7N-5A9, Canada; § Department of Physics, Muthurangam Government Arts and Science College (Autonomous), Vellore, Affiliated to Thiruvalluvar University, Serkadu, Vellore 632115, Tamil Nadu, India; ∥ Department of Data Science and Computer Applications, Manipal Institute of Technology, Manipal Academy of Higher Education, Manipal 576104, Karnataka, India

## Abstract

Lanthanum oxide (La_2_O_3_) nanoparticles stand out as promising photocatalysts due to their remarkable stability and photocatalytic properties. In this study, La_2_O_3_ nanoparticles were synthesized via a hydrothermal method and explored how varying calcination time (3 and 5 h) influences their structural, morphological, optical, and catalytic properties. X-ray diffraction (XRD) confirmed stable hexagonal structure, with crystallite sizes increasing from 32.79 to 45.49 nm, while UV–vis absorption studies revealed that increasing calcination time led to a gradual decrease in bandgap energy from 4.6 to 4.4 eV, making the material more effective at utilizing light for pollutant degradation. When tested against a range of organic dyes, La_2_O_3_ nanoparticles calcinated for 5 h exhibited the highest degradation efficiencies, due to their improved crystallinity and enhanced charge carrier movement. The photocatalytic process followed first-order kinetics, and recyclability tests showed that the nanoparticles retained their efficiency over multiple cycles. Radical scavenger tests confirmed that hydroxyl radicals (^•^OH) and superoxide radicals (^•^O_2_^–^) were the dominant reactive species involved in dye degradation, affirming the key mechanism behind the observed photocatalytic performance. These results highlight how fine-tuning calcination time can significantly enhance La_2_O_3_’s potential, making it an eco-friendly solution for wastewater treatment.

## Graphical abstract

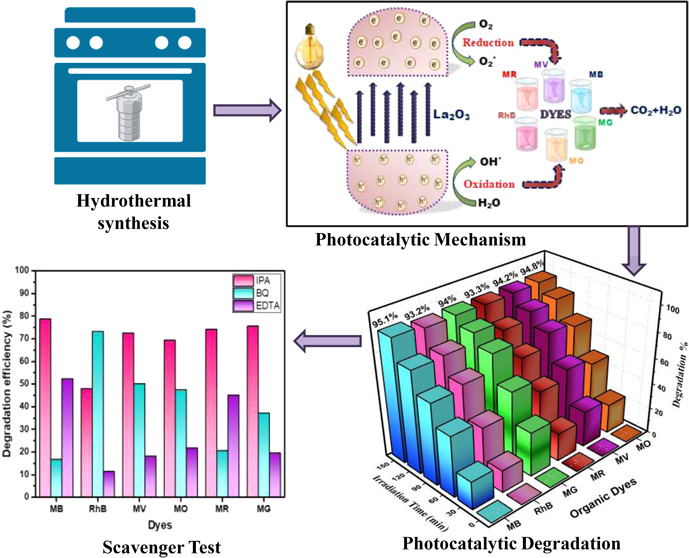

## Introduction

The field of nanotechnology has undergone remarkable growth, offering innovative solutions across numerous disciplines, particularly in environmental science. Photocatalysis, a light-driven process, has emerged as a groundbreaking technology for addressing water pollution, one of the most critical environmental challenges of modern times. Industrial wastewater is frequently laden with hazardous pollutants, including synthetic dyes, pharmaceuticals, and heavy metals, which can severely impact aquatic life and pose risks to human health. Conventional treatment techniques, such as chemical oxidation, adsorption, and biological methods, often struggle with challenges related to efficiency, large-scale application, and economic feasibility.^1,2^ Consequently, advanced oxidation processes (AOPs) have gained traction, with photocatalysis being a standout due to its capability to utilize light energy to break down pollutants into nontoxic byproducts, promoting a shift toward green and sustainable environmental practices.^3,4^

Nanomaterials have revolutionized the field of photocatalysis by offering exceptional properties, including large surface areas, tunable electronic structures, and efficient charge transport. These characteristics enable optimization of light absorption and charge carrier dynamics, thereby enhancing the photocatalytic breakdown of pollutants. Semiconductor nanoparticles, in particular, are at the forefront of this innovation. Their ability to generate reactive oxygen species (ROS) under light exposure allows them to degrade harmful organic compounds effectively, making them a critical component in modern wastewater treatment technologies.^5,6^

Lanthanum oxide, a rare-earth-metal oxide, has gained significant attention as a photocatalyst due to its remarkable characteristics. Its high chemical and thermal stability, wide bandgap, and cost-effectiveness make it a promising material for photocatalytic applications, offering an alternative to conventional options.^7,8^ Additionally, the unique electronic structure of lanthanum facilitates improved charge separation and reduces electron–hole recombination, enhancing the photocatalytic efficiency. These attributes, combined with its strong affinity for adsorbing pollutant molecules, position La_2_O_3_ as a robust candidate for addressing water pollution challenges in a sustainable and economical manner.^9^

The increasing pressures of population growth, industrial development, and climate change have amplified concerns surrounding water scarcity and pollution, particularly in developing regions. Industrial sectors, such as textile manufacturing, contribute significantly to this issue by discharging untreated dye-laden wastewater, which contaminates water resources. This escalating problem highlights the urgent need for sustainable and efficient wastewater management techniques.^10^ Photocatalysis, leveraging the unique capabilities of nanomaterials such as La_2_O_3_, provides a transformative solution. By degrading harmful organic dyes and other pollutants, photocatalysts derived from La_2_O_3_ provide an environmentally friendly, energy-efficient, and economical solution for treating industrial wastewater, contributing to the advancement of sustainable water resource management.^10,11^

## Experimental Section

### Materials

La_2_O_3_ NPs were synthesized using lanthanum­(III) nitrate hexahydrate (La­(NO_3_)_3_·6H_2_O) and ammonium hydroxide (NH_4_OH) as precursors. Ethanol and double-distilled water (DD) were utilized as washing agents and solvents throughout the procedure. All of the ingredients, including AR-grade chemicals, were bought from Merck, India.

### Synthesis of La_2_O_3_ Nanoparticles

The synthesis of La_2_O_3_ nanoparticles was carried out by separately dissolving 0.1 mol of lanthanum nitrate (La­(NO_3_)_3_) and 0.1 mol of hexamethylenetetramine (HMTA) in double-distilled water. The two solutions were then combined and stirred thoroughly. The pH of the solution was adjusted to 12 using liquid ammonia (NH_4_OH). The mixture was autoclaved at 180 °C for 24 h and then cooled to room temperature. The precipitate was filtered, washed with distilled water and ethanol, and dried at 80 °C for 3 h. The dried material was then calcined at 900 °C for 3 h to enhance its crystallinity. An additional sample was prepared by repeating the synthesis process with a calcination duration of 5 h to investigate the impact of calcination time on the material’s properties.

### Experiment Procedure for Photocatalytic Activity

The photocatalytic behavior of La_2_O_3_ nanoparticles was thoroughly investigated against various organic dyes, including methylene blue (MB), rhodamine B (RhB), malachite green (MG), methyl red (MR), methyl violet (MV), and methyl orange (MO), under light irradiation at room temperature. The experiment commenced with the preparation of dye solutions, where 0.1 g of each dye was dissolved in 100 mL of distilled water, resulting in solutions with distinct colors that allowed for precise optical analysis. These stock solutions were then diluted to a working concentration of 10 ppm, which was used for the photocatalytic experiments. This concentration range falls within the linear absorbance region of each dye, as confirmed through calibration plots, ensuring compliance with Beer–Lambert’s law (*R* ^2^ > 0.99 for all dyes).

In the initial adsorption phase, 0.1 g of La_2_O_3_ nanoparticles was dispersed into 100 mL of the 10 ppm MB dye solution. The mixture was stirred continuously in the dark for 1 h to establish adsorption–desorption equilibrium between the dye molecules and the nanoparticle surfaces. During this time, dye molecules adhered to the La_2_O_3_ surface through adsorption processes, balancing the rates of adsorption and desorption. This equilibrium ensured uniform surface interaction, optimizing the nanoparticles’ catalytic readiness.

After the adsorption step, the solution was transferred into a photochemical reactor equipped with a dual-jacketed beaker designed to regulate the temperature during the reaction. Continuous stirring ensured homogeneous mixing, as the reactor was irradiated with halogen light. The degradation experiment was carried out over 150 min, with 5 mL aliquots taken every 30 min for spectroscopic analysis. The double-jacketed design of the reactor facilitated temperature stability by the circulation of cool water throughout the process. The degradation progress of the dyes was tracked using a UV–vis spectrophotometer over the wavelength range of 200–800 nm. A consistent decrease in absorbance at the characteristic wavelengths of the dyes reflected their breakdown. This degradation was attributed to the interaction between the dyes and the light-activated La_2_O_3_ nanoparticles, which generated reactive species capable of decomposing the dye molecules into less harmful components.^12–14^

The same experimental protocol was applied to the other dyes (RhB, MG, MR, MV, and MO) to validate the photocatalytic efficiency of La_2_O_3_ nanoparticles across a range of organic pollutants. The results demonstrated significant degradation of all tested dyes, underscoring the potential of La_2_O_3_ nanoparticles as highly effective materials for tackling water pollution and advancing environmental remediation strategies.

## Results and Discussion

### Structural Analysis

The structural properties of La_2_O_3_ nanoparticles synthesized at different calcination times (3 and 5 h) were carefully studied using X-ray diffraction (XRD), a key technique for analyzing crystallite size and structural characteristics. Figure 1(a) shows the XRD patterns of the nanoparticles calcined for these durations, with distinct diffraction peaks confirming the hexagonal crystal structure of La_2_O_3_ as shown in Figure 1(b) and validated by JCPDS card no. 74–2430. These peaks closely match reported values in the literature, affirming the phase purity and high crystallinity of the synthesized nanoparticles.

**Figure 1:**
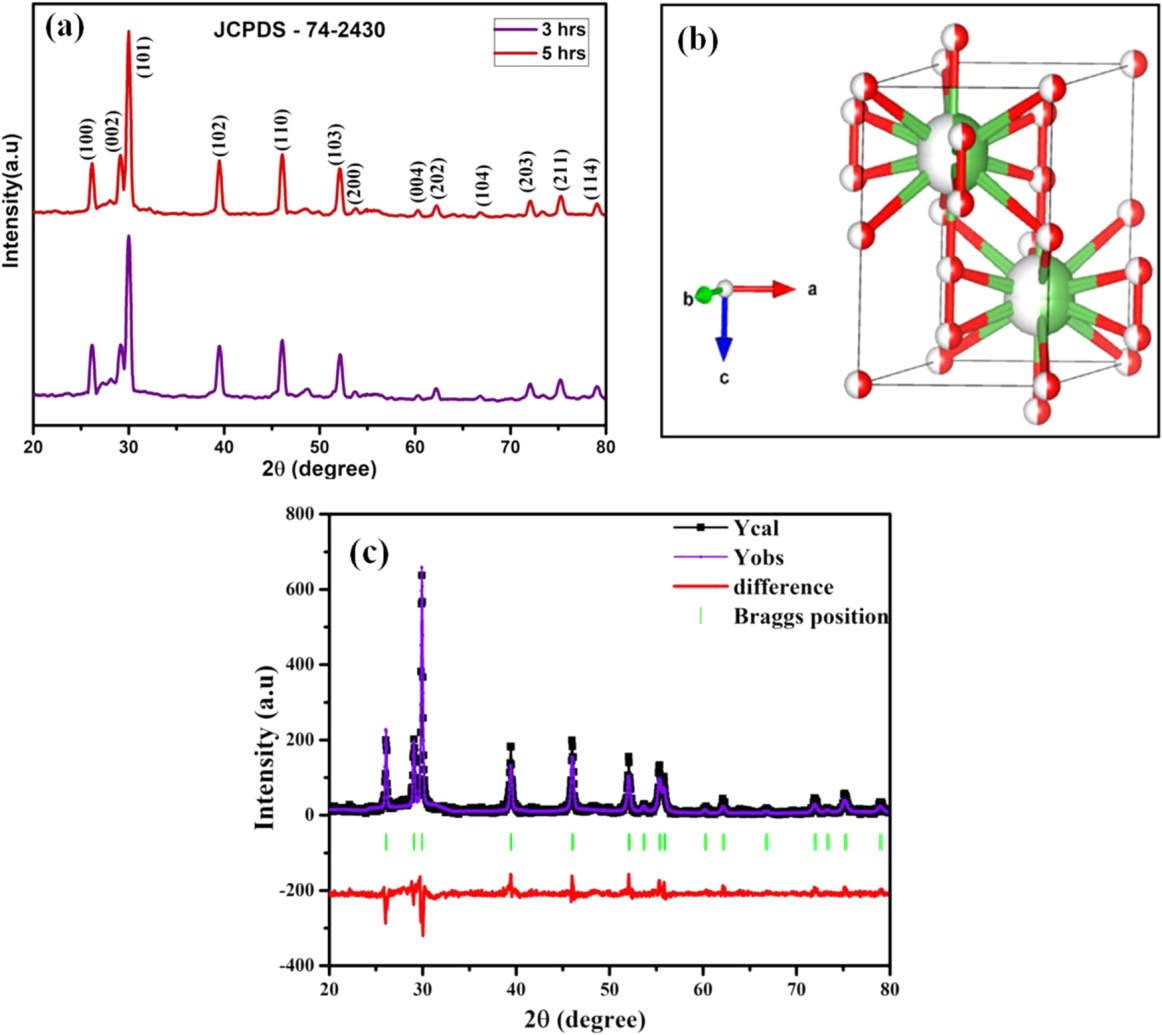
(a) XRD patterns of La_2_O_3_ nanoparticles synthesized at various calcination times, (b) structure of La_2_O_3_, and (c) refinement plot of La_2_O_3_ nanoparticles calcinated for 5 h.

The crystallite sizes, calculated using the Scherrer equation,^15,16^ were found to increase with calcination time, measuring 32.79 and 45.49 nm for 3 and 5 h, respectively. The detailed refinement parameters, including lattice constants, *R*-factor values, and unit cell dimensions, are summarized in Table 1. These parameters were obtained through Rietveld refinement of the XRD data using FullProf Suite software, with the refinement results presented in Figure 1(c). The data confirms the stability of the hexagonal phase across all samples, showing no significant phase transitions with longer calcination time.

**Table 1:** Refinement Parameters of La_2_O_3_ Nanoparticles Calcinated for 5 h

sample	La_2_O_3_
crystal system	hexagonal
lattice parameters	*a* = 4.057, *b* = 4.057, *c* = 6.43
	α = 90°, β = 90°, γ = 120°
unit cell volume	91.654
GoF	1.6

The increase in crystallite size with prolonged calcination is attributed to processes like grain coarsening (Ostwald ripening), where increased thermal energy enhances atomic diffusion, causing smaller grains to merge into larger ones.^17,18^ Additionally, extended heat treatment reduces lattice defects such as dislocations and grain boundaries, allowing the material to achieve a more stable lower-energy state. The sintering effect, driven by surface and volume diffusion during prolonged heating, further promotes nanoparticle growth.^19^

To further validate the structural analysis, the W–H method was applied to study the La_2_O_3_ nanoparticles calcinated for 3 and 5 h. This method provides a detailed understanding by separating the contributions of crystallite size and lattice strain to peak broadening in the XRD patterns. In this approach, the *X*-axis represents 4 sin θ, while the *Y*-axis represents βcosθ. Figure 2 displays the W–H plots for the nanoparticles calcined at each duration. The crystallite sizes determined from these plots are compared with those obtained by using the Debye–Scherrer equation, as summarized in Table 2. This analysis further supports the findings, providing a comprehensive assessment of the structural properties of the La_2_O_3_ nanoparticles.

**Figure 2:**
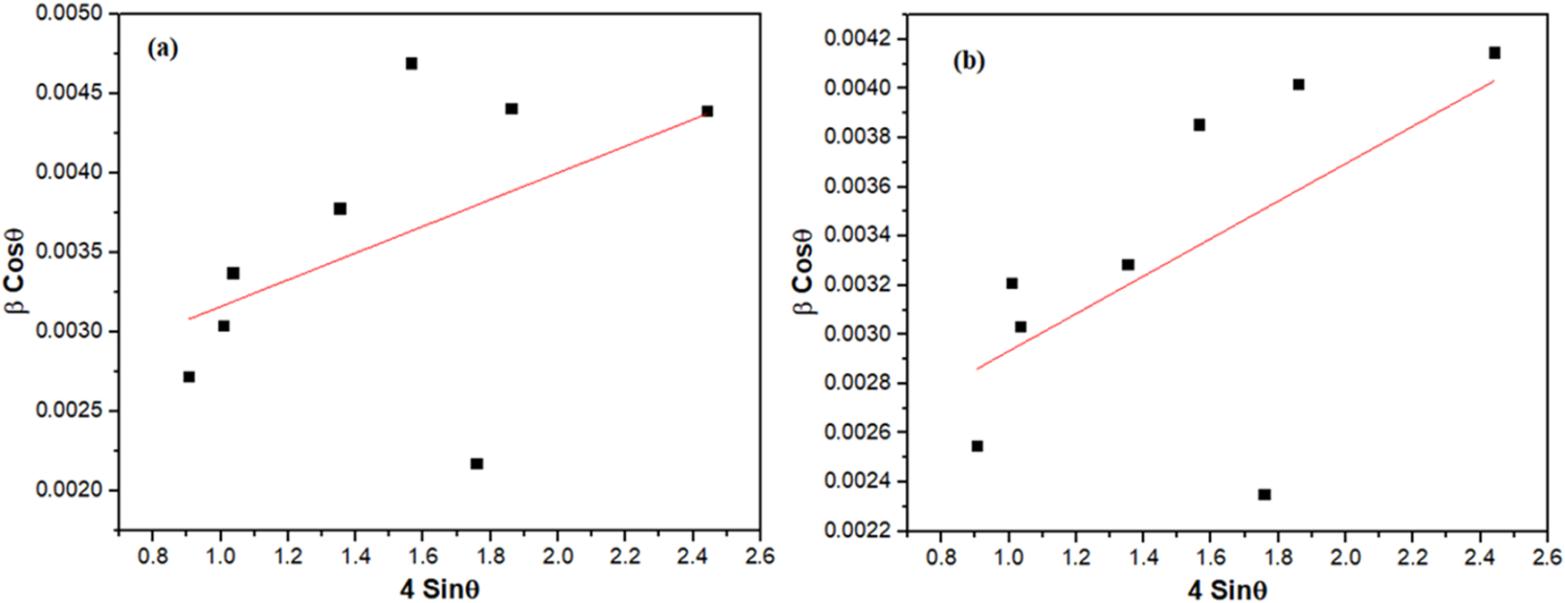
W–H plot of La_2_O_3_ nanoparticles calcinated for (a) 3 h and (b) 5 h.

**Table 2:** List of Structural Parameters of Synthesized La_2_O_3_ Nanoparticles

	crystallite size (nm)			
calcination time (h)	Scherrer equation	W–H plot	dislocation density (δ) nm^–2^	microstrain (ε)
3	32.79	36.34	0.000929	0.001143
5	45.49	49.22	0.000483	0.000826

In addition to crystallite size, microstrain (∈) and dislocation density (δ) were calculated using eqs 1 and 2

1 microstrain,∈=β⁡cos⁡θ/4

2 $$\mathrm{dislocation}\;\mathrm{density},\delta =1/D^{2}\;{\left(\mathrm{nm}\right)}^{-2}$$

The analysis revealed that increasing calcination time led to significant growth in crystallite size along with a noticeable reduction in microstrain and dislocation density, as illustrated in Figure 3. This trend indicates that prolonged calcination enhances crystallinity and reduces lattice distortions.^20^ The decrease in microstrain and dislocation density reflects a more ordered lattice structure, as extended thermal treatment helps eliminate internal defects by providing the energy needed for structural relaxation.^21^ The W–H plot analysis further validated the crystallite size and microstrain values derived from the XRD data, confirming the high crystallinity and phase purity of the La_2_O_3_ nanoparticles. The results consistently aligned with the expected hexagonal structure. By integrating the Debye–Scherrer equation with W–H analysis, a clearer understanding of the effects of calcination time on crystallite size, microstrain, and dislocation density was achieved.

**Figure 3:**
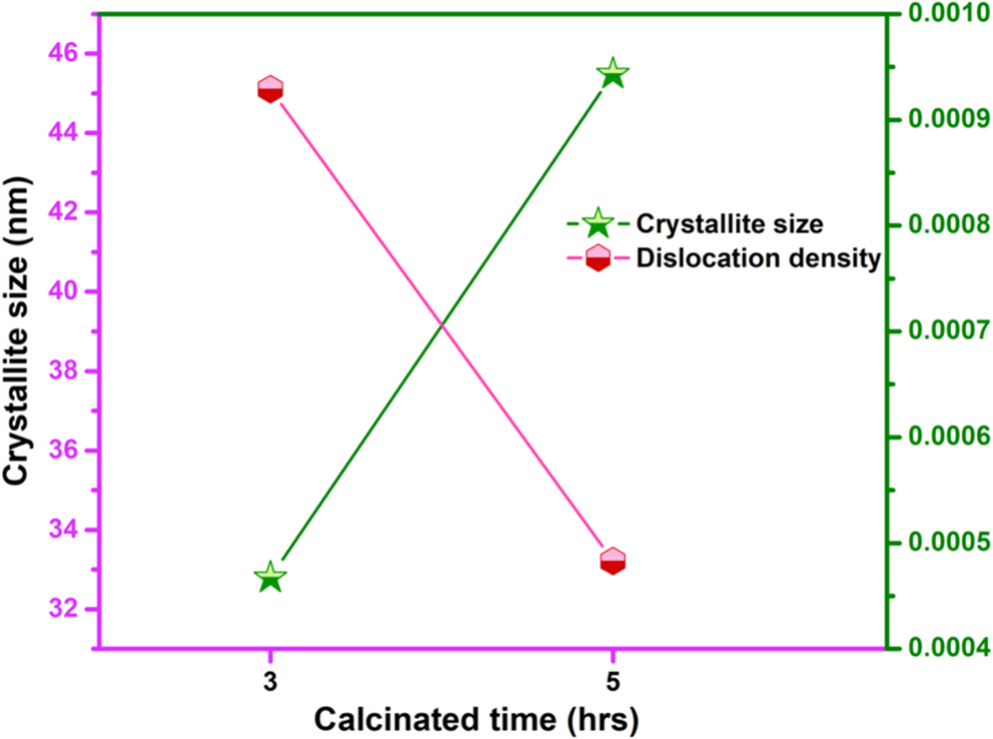
Crystallite size and dislocation density for various calcination times.

The slight differences in crystallite size values between the Scherrer equation and the W–H method can be attributed to their distinct approaches. The Scherrer equation considers peak broadening to result exclusively from crystallite size, while the W–H method considers both crystallite size and strain effects. This makes the W–H method a more precise tool, especially for materials in which lattice strain significantly contributes to peak broadening. As a result, the W–H analysis not only provides a more accurate estimation of crystallite size but also offers deeper insights into the structural properties of La_2_O_3_ nanoparticles calcinated for different durations.^22,23^

### UV–Vis Absorption Spectroscopy Analysis

The bandgap energies of the nanoparticles were determined from Tauc plots (Figure 4) using UV–vis absorption spectroscopy. The absorption spectrum revealed a gradual increase in intensity as the calcination time was extended, suggesting better light absorption. At the same time, the optical bandgap energies showed a consistent decrease, measured at 4.6 and 4.4 eV for nanoparticles calcinated at 900 °C for 3 and 5 h, respectively. This reduction in bandgap energy can be explained by structural and electronic improvements that occur during prolonged calcination.^24^ The extended heating at high temperatures enhances the crystalline structure by relieving lattice strain and eliminating defects like dislocations and vacancies, which can create energy states within the bandgap.^25,26^ These defects usually hinder the movement of charge carriers and encourage recombination. With fewer defects, the nanoparticles achieve a more consistent and stable electronic configuration.

**Figure 4:**
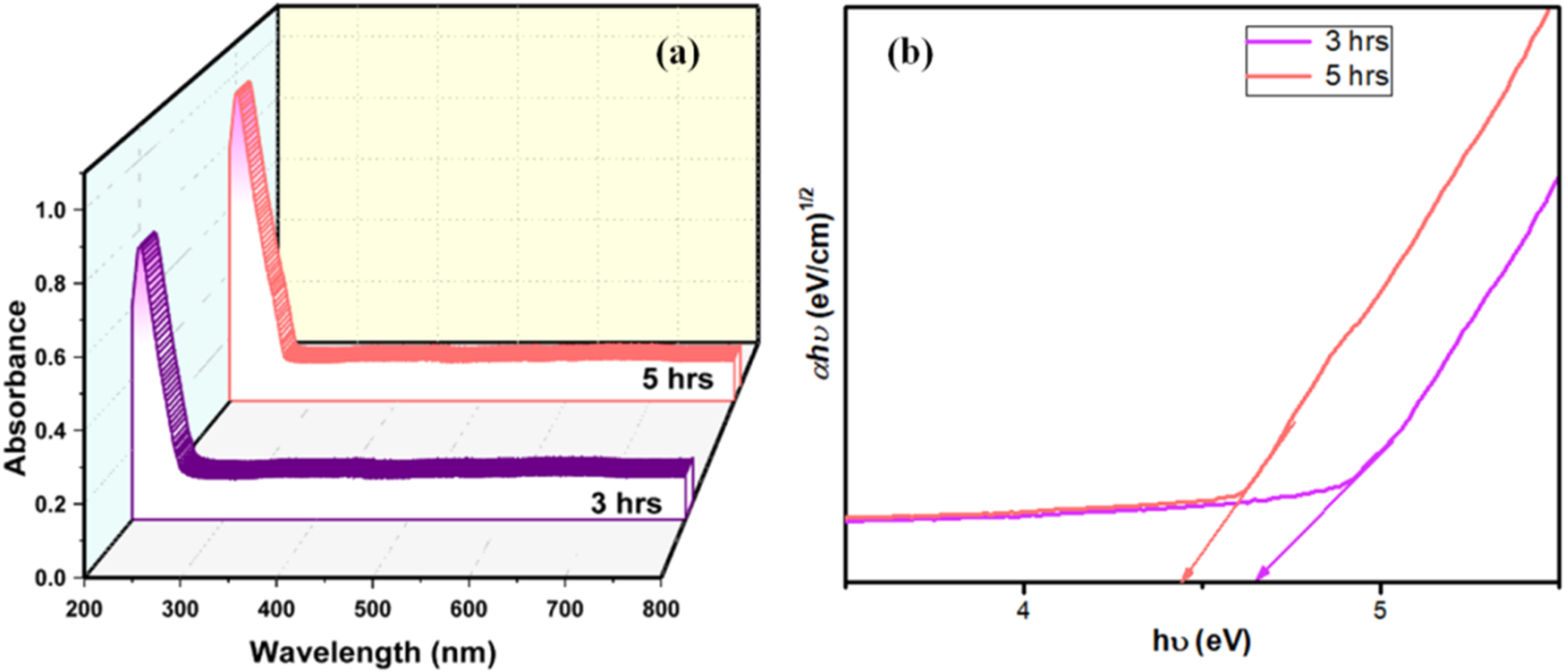
(a) UV–vis absorption spectra of La_2_O_3_ nanoparticles and (b) Tauc’s plot.

Additionally, the extended calcination process promotes thermal annealing, which increases orbital overlap and modifies the electronic structure, resulting in a shift in the band edges and a narrower bandgap.^25^ The enhanced light absorption and reduced bandgap energy observed in these nanoparticles make them promising candidates for photocatalytic applications. These changes enable the material to absorb a broader spectrum of sunlight, improving its performance in processes, such as energy conversion and pollutant breakdown. This study highlights how adjusting the calcination conditions can optimize the optical and electronic properties of nanoparticles, opening new opportunities for their use in environmentally sustainable technologies.

### FTIR Analysis

Figure 5 presents the FTIR spectra of La_2_O_3_ nanoparticles calcinated for 3 and 5 h, providing insights into how calcination temperature impacts their molecular structure and bonding. The spectra reveal prominent absorption bands at 3610 ^27^ and 3443 cm^–1^,^28^ which are linked to O–H stretching vibrations. These bands suggest the presence of water molecules adsorbed during sample preparation. Additionally, a distinct peak at 1632 cm^–1^,^29^ corresponding to O–H bending vibrations, further confirms the retention of moisture in the samples. The band at 1386 cm^–1^ is associated with the asymmetric deformation of NH_4_^+^ ions.^30^ Meanwhile, peaks at 2920, 2850, and 1476 cm^–1^ indicate C–H stretching vibrations,^30^ likely originating from organic residues left behind during synthesis. Characteristic peaks observed at 857,^30^ 643,^27–31^ and 496 cm^–1^ ^32^ are attributed to La–O stretching and bending vibrations, confirming the formation of La_2_O_3_ nanoparticles. These findings highlight how the calcination time influences the molecular and chemical composition of the nanoparticles. FTIR proves to be an invaluable tool for identifying functional groups and understanding the chemical properties of thermally treated materials.

**Figure 5:**
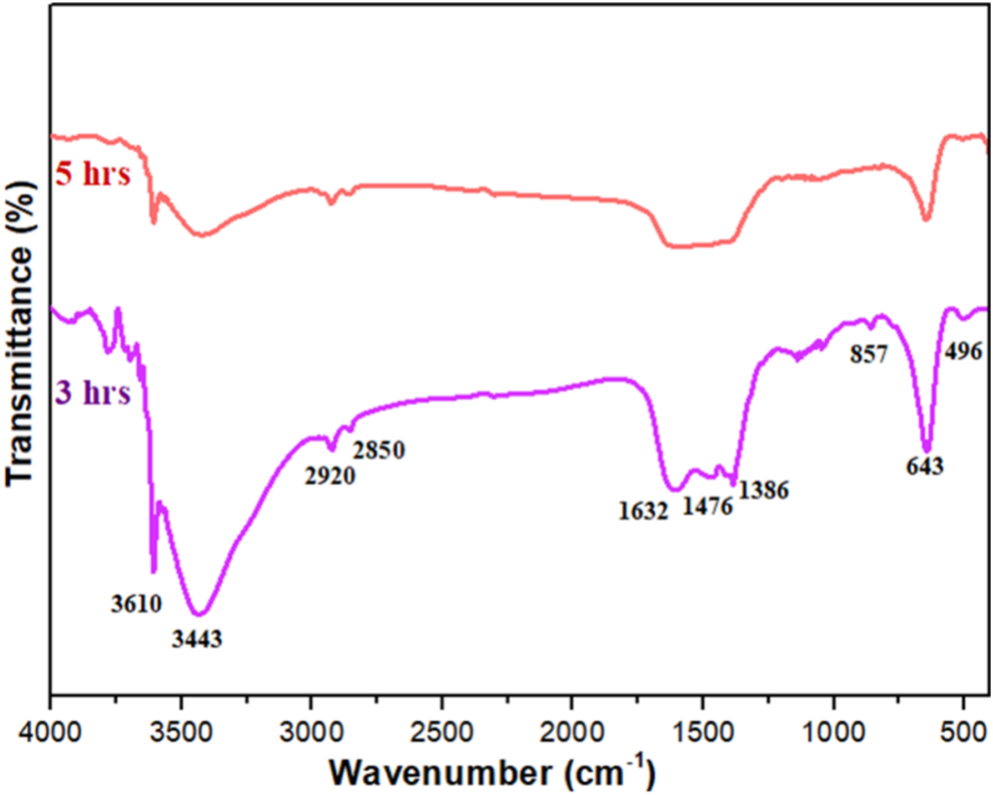
FTIR spectra of La_2_O_3_ nanoparticles.

### TEM Analysis

Figure 6(a,c) presents the TEM analysis of La_2_O_3_ nanoparticles, revealing a consistent spherical shape across all samples. The analysis also shows that particle size increases with longer calcination times.^33^ The calculated average particle sizes were 72 and 85 nm for calcination times of 3 and 5 h, respectively. This gradual growth in particle size can be attributed to thermally driven processes such as Ostwald ripening.^34^ During calcination, higher temperatures provide sufficient energy for atoms to diffuse more effectively, leading to the dissolution of smaller particles and their redeposition onto larger ones to reduce surface energy.^35^ Additionally, the reduction of surface defects, including vacancies and dislocations, allows for better structural reorganization and the expansion of crystalline domains, further contributing to particle growth.^36,37^ These findings highlight how calcination time plays a key role in determining particle size and morphology, which are crucial factors influencing the functional properties of La_2_O_3_ nanoparticles. Fine-tuning calcination conditions is essential for tailoring these nanoparticles to meet the requirements of specific applications, particularly in areas where size-dependent properties are critical for performance.

**Figure 6:**
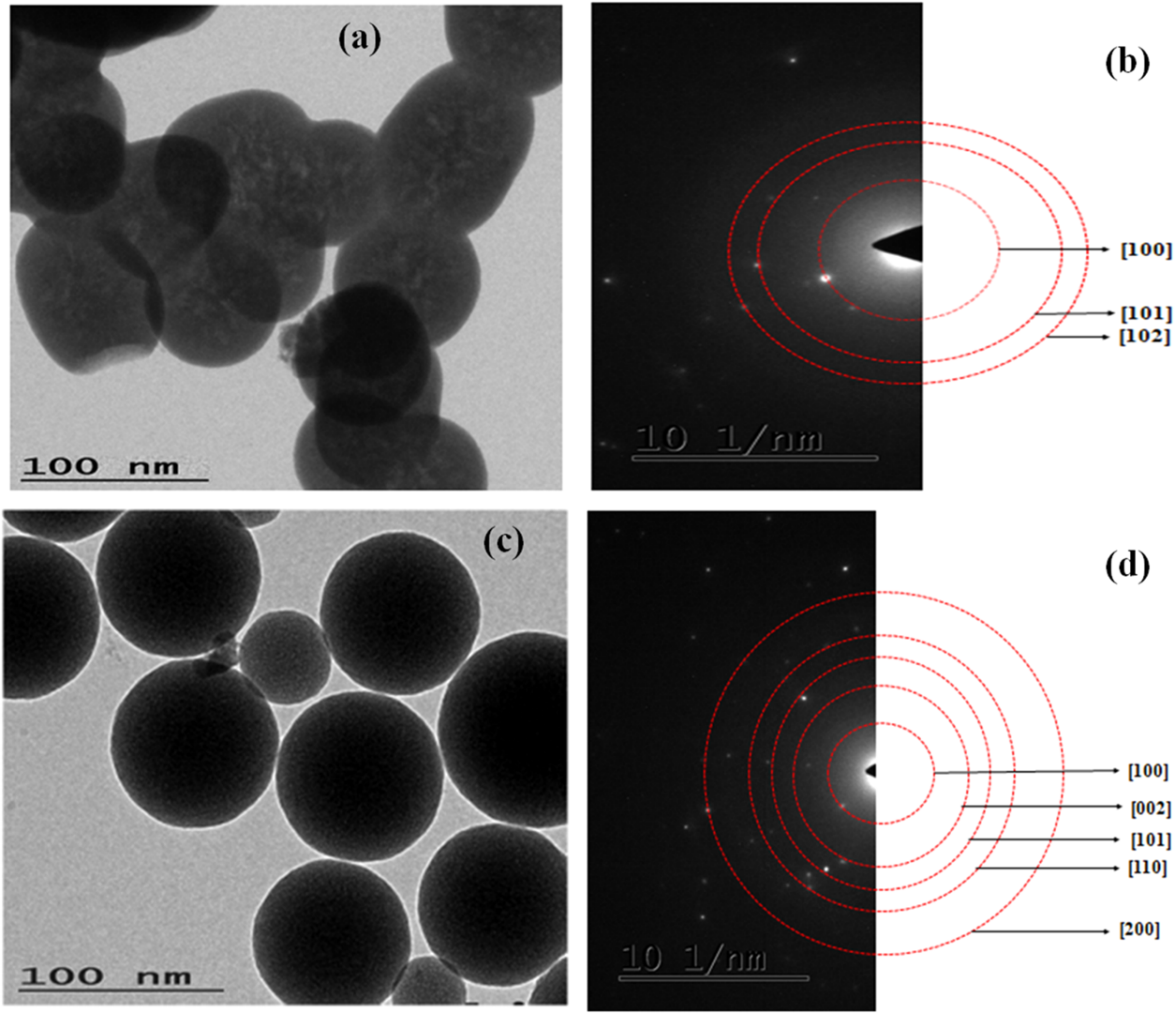
TEM images of La_2_O_3_ nanoparticles calcinated for (a) 3 h and (c) 5 h and along with the corresponding SAED pattern (b) 3 h and (d) 5 h.

In addition to morphological analysis, the selected area electron diffraction (SAED) patterns (Figure 6(b),(d)) further support the crystallinity of the La_2_O_3_ nanoparticles. The sample calcined for 3 h exhibits broad but discernible diffraction rings, indicating the presence of nanocrystalline domains. After 5 h of calcination, the SAED pattern reveals intense and distinct rings, confirming enhanced crystallinity and well-developed lattice planes. These diffraction patterns align with the XRD results and confirm that the increased calcination time promotes both particle growth and improved structural order. Together, the TEM and SAED findings demonstrate how controlling the calcination parameters is key to optimizing the physical properties of La_2_O_3_ nanoparticles for high-performance applications.

## Photocatalytic Activity

### Photocatalytic Mechanism

Figure 7 illustrates the experimental process used to study the photocatalytic behavior of La_2_O_3_ nanoparticles. The mechanism begins when light interacts with the nanoparticles dispersed in the dye solution. This interaction excites electrons from the valence band to the conduction band, leaving holes in the valence band. The resulting electron–hole pairs are vital for driving photocatalytic reactions. At the surface of the nanoparticles, these charge carriers engage in oxidation and reduction processes, leading to the production of reactive oxygen species (ROS) such as hydroxyl radicals (^•^OH) and superoxide anions (O_2_^–^). These ROS are highly effective in degrading the organic pollutants present in the dye solution. Under visible light, La_2_O_3_ nanoparticles demonstrate strong photocatalytic activity due to their ability to generate a significant amount of ROS.

**Figure 7:**
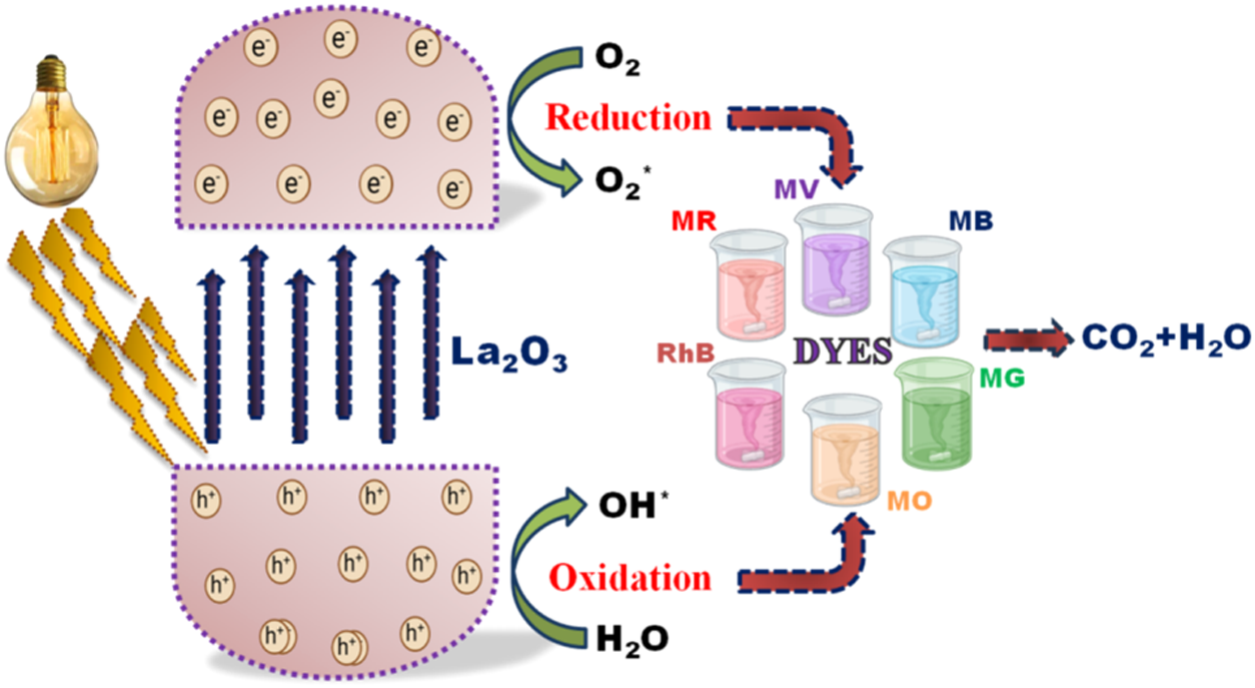
La_2_O_3_ nanoparticle’s photocatalytic mechanism.

These reactive species interact with the dye molecules, breaking down their complex structures into simpler, harmless products such as carbon dioxide and water. Factors such as the nanoparticle surface area, intensity of the light source, and duration of exposure play crucial roles in determining the efficiency of this degradation process. The photocatalytic performance of La_2_O_3_ nanoparticles is typically evaluated by monitoring the reduction in dye concentration over time using UV–vis spectroscopy. The findings confirm that La_2_O_3_ nanoparticles are effective in reducing dye concentrations upon exposure to visible light, showcasing their potential in environmental cleanup efforts.

Their ability to produce reactive oxygen species makes them an excellent choice for water purification applications.^38–40^ Further advancements in the synthesis and optimization of these nanoparticles could enhance their photocatalytic properties, making them even more efficient for tackling environmental issues, such as water pollution.

### Photocatalytic Performance

The photocatalytic activity of La_2_O_3_ nanoparticles calcinated for varying times (3 and 5 h) was evaluated using UV–vis absorption spectra for different dyes, including MB, RhB, MG, MR, MV, and MO which exhibit their characteristic absorption peaks at 664, 553, 618, 522, 573, and 470 nm, respectively. During light exposure, each dye solution showed a gradual reduction in the maximum absorption peak intensity, signifying the progressive degradation of the dye molecules, as depicted in Figure 8. The process was monitored at 30 min intervals for a total duration of 150 min, at which point the absorbance of each dye solution approached zero, rendering the solutions nearly colorless and indicating near-complete degradation of the dyes.

**Figure 8:**
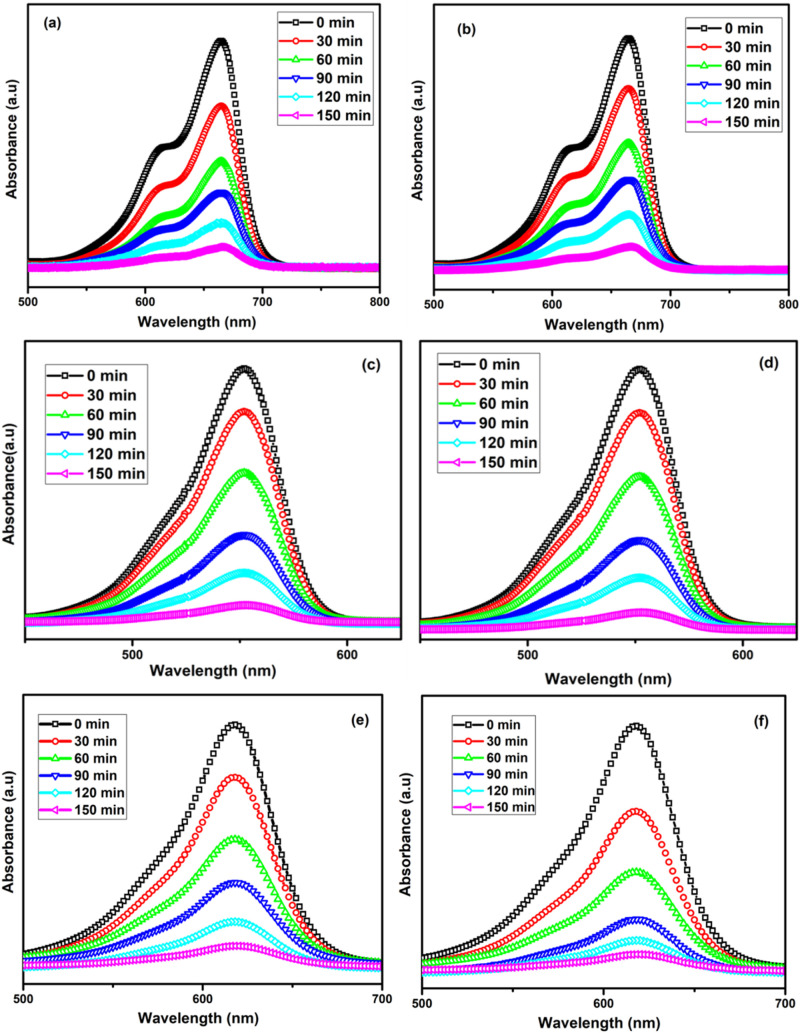

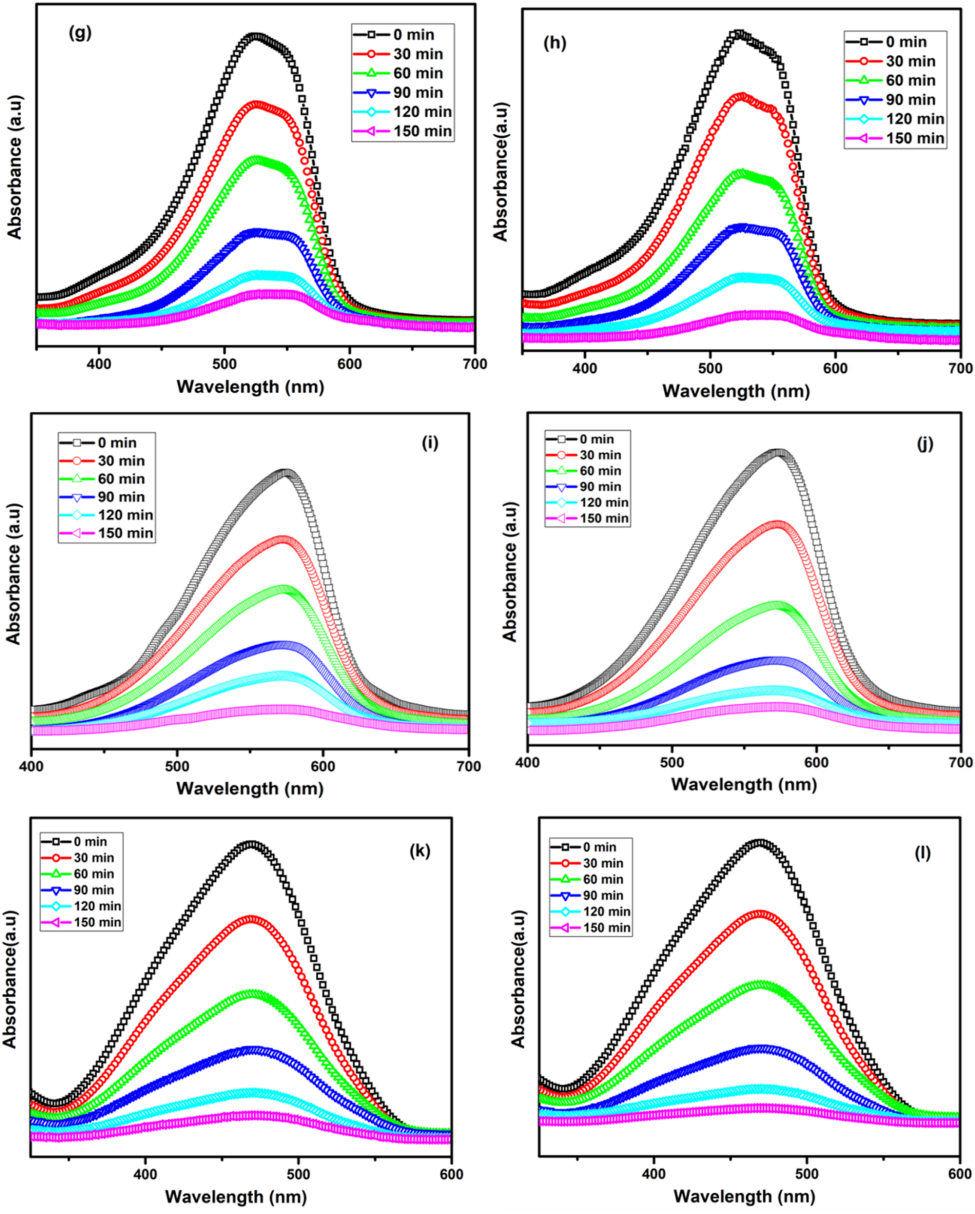
Degradation of MB (a, b), RhB (c, d), MG (e, f), MR (g, h), MV (i, j), and MO (k, l) dyes for the prepared La_2_O_3_ nanoparticles calcinated for 3 and 5 h.

The photodegradation efficiency can be determined using eq 3.

3 $$\mathrm{photodegradation}\;\mathrm{efficiency}=\frac{C_{0}-C_{t}}{C_{0}}\times 100\%$$

where (*C*_0_) represents the initial dye concentration and (*C_t_*) is the concentration at a specific time during the photodegradation process.^38–40^ The calculated efficiencies revealed that La_2_O_3_ nanoparticles calcined for longer durations exhibited an enhanced photocatalytic performance, as shown in Figure 9.

**Figure 9:**
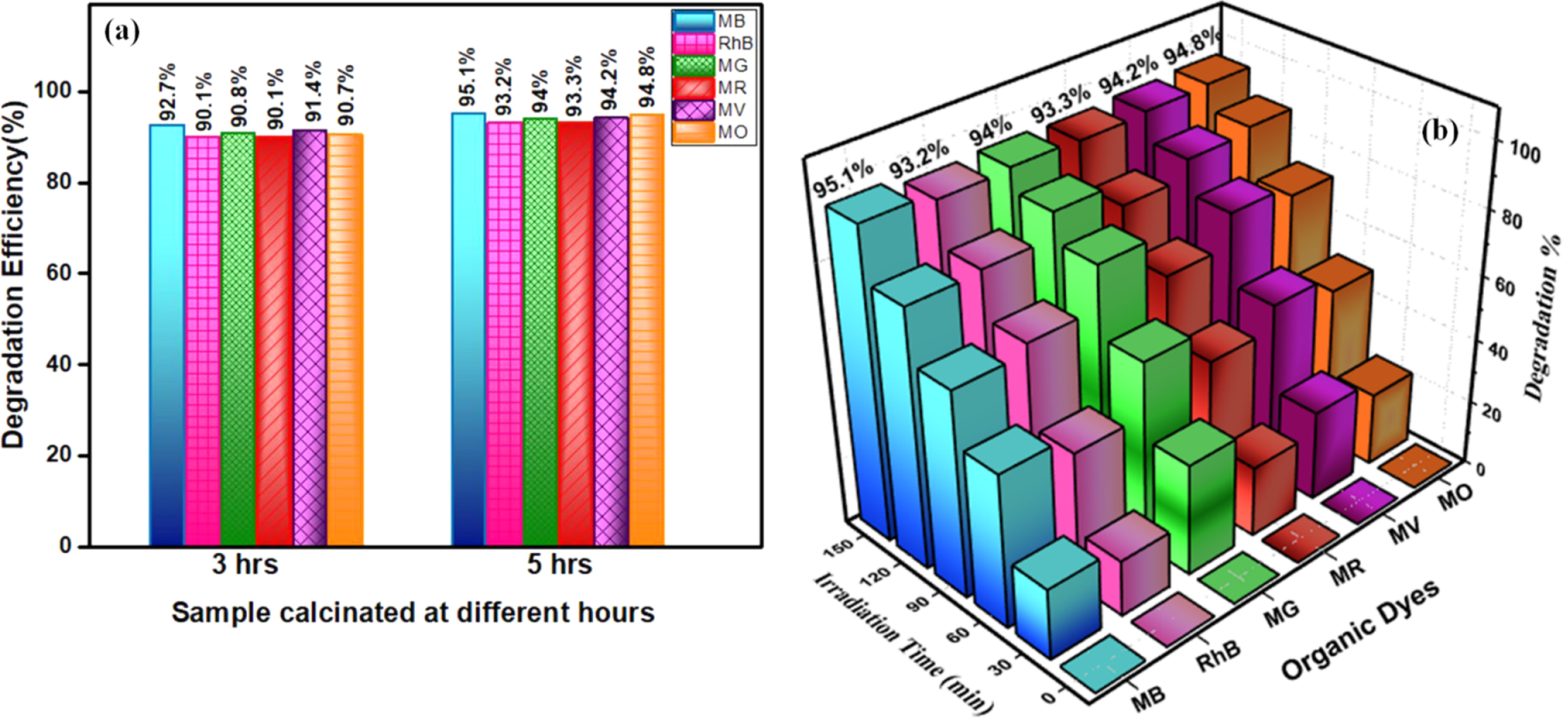
(a) Degradation efficiency of La_2_O_3_ nanoparticles calcinated for 3 and 5 h toward various organic dyes. (b) Relationship between irradiation time and degradation percentage obtained for various dyes with La_2_O_3_ nanoparticles calcinated for 5 h.

For instance, the degradation efficiencies for MB were 92.7 and 95.1% for nanoparticles calcined for 3 and 5 h, respectively. Similar trends were observed for other dyes: RhB achieved 90.1 and 93.2%; MG achieved 90.8 and 94%; MR achieved 90.1 and 93.3%; MV achieved 91.4 and 94.2%; and MO achieved 90.7 and 94.8% degradation for the respective calcination times.

A comparison of the photocatalytic efficiencies of various metal oxides already reported in the literature is presented in Table 3. However, the synthesized La_2_O_3_ demonstrated a photocatalytic efficiency of up to 95.1% under halogen light, highlighting its strong potential for photocatalytic applications and aligning well with findings reported in the literature.

**Table 3:** Comparison of Photocatalytic Efficiency of La_2_O_3_ with Previously Reported Literature

photocatalyst	target dye	light source	degradation efficiency	time (min)	refs
TiO_2_	methylene blue	UV	88%	180	^41^
ZnO	rhodamine B	UV	85%	150	^42^
Fe_3_O_4_/TiO_2_/CuO	malachite green	visible light	60%	150	^43^
WO_3_	methyl orange	visible light	18%	90	^44^
La_2_O_3_ (this work)	MB, RhB, MG, MR, MV, and MO	halogen light	up to 95.1% (MB)	150	current study

The improved photocatalytic performance with a longer calcination time is likely due to enhanced crystallinity of La_2_O_3_ nanoparticles, as confirmed by XRD analysis. Longer calcination times reduce lattice defects and dislocations, leading to fewer recombination sites for photogenerated charge carriers, thereby increasing the efficiency of charge separation. Additionally, a higher degree of crystallinity provides better photon absorption and improved electron transport, both of which contribute to the superior photocatalytic degradation observed for nanoparticles calcinated for extended durations.

### Kinetic Study

La_2_O_3_ nanoparticles (calcinated for 5 h) exhibited photocatalytic activity, following first-order kinetics as indicated by the linear slope in ln­(*C*_0_/*C_t_*) vs time plot (Figure 10). This linearity confirms that the degradation rate is directly proportional to the reduction of the residual dye in solution, which is characteristic of first-order reaction behavior. The calculated rate constants for the photodegradation of each dye highlight the efficiency of La_2_O_3_ nanoparticles as photocatalysts. Specifically, the values for MB, RhB, MG, MR, MV, and MO dyes were 1.86 × 10^–2^, 1.68 × 10^–2^, 1.82 × 10^–2^, 1.73 × 10^–2^, 1.88 × 10^–2^, and 1.89 × 10^–2^ min^–1^, respectively. Among the tested dyes, MB exhibited the highest degradation rate, reflecting the remarkable photocatalytic capability of La_2_O_3_ nanoparticles.^38–40,45,46^

**Figure 10:**
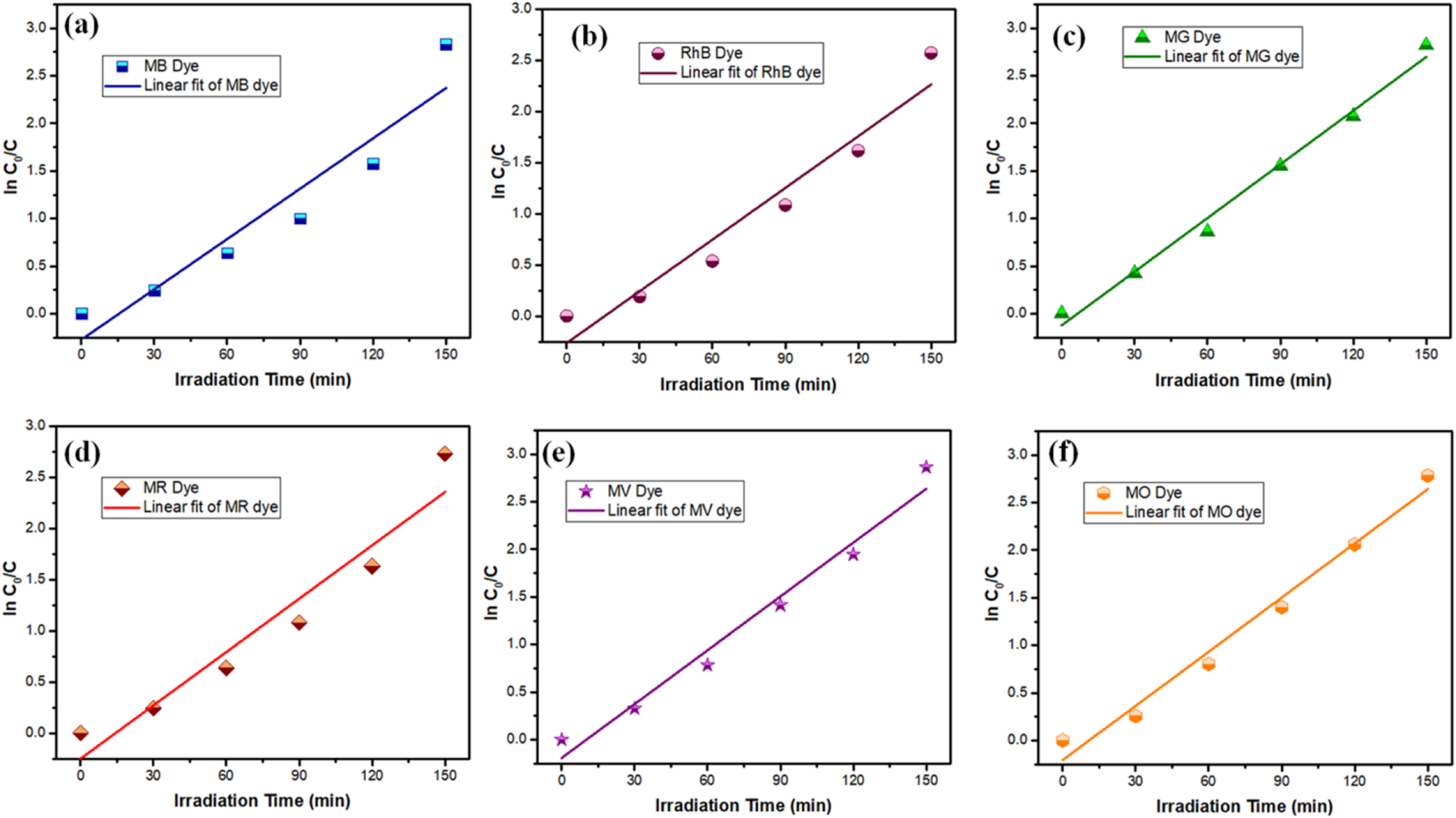
Plot of ln­(*C*_0_/*C_t_*) versus irradiation time for (a) MB, (b) RhB, (c) MG, (d) MR, (e) MV, and (f) MO dyes for La_2_O_3_ nanoparticles calcinated for 5 h.

This impressive performance can be attributed to the inherent properties of La_2_O_3_ nanoparticles, including their high surface area and optimized bandgap. Under light exposure, electron–hole pairs initiate redox reactions, breaking down organic dyes into smaller, less harmful compounds. The first-order kinetics observed in these experiments underscores the robust photocatalytic activity of La_2_O_3_ nanoparticles. Their ability to effectively degrade diverse dyes demonstrates their potential as a versatile and powerful photocatalyst for treating wastewater contaminated with organic dyes. These findings highlight the significant role of La_2_O_3_ nanoparticles in promoting sustainable water purification processes and ensuring access to clean water resources.

### Recyclability Study

Figure 11 illustrates the reusability of La_2_O_3_ nanoparticles, calcinated for 5 h, tested over three cycles to evaluate their efficiency in degrading dyes such as MB, RhB, MG, MR, MV, and MO dyes. After each cycle, the nanoparticles were recovered, cleansed with distilled water and ethanol to remove residues, and dried for further use. The results showed excellent performance in the first cycle, with only a slight decline in efficiency over the next two cycles. Even in the third cycle, the nanoparticles maintained a consistently high level of activity, demonstrating their durability and stability. These findings highlight the significant potential of La_2_O_3_ nanoparticles as reusable photocatalysts for environmental pollutant remediation.^38–40,45–47^

**Figure 11:**
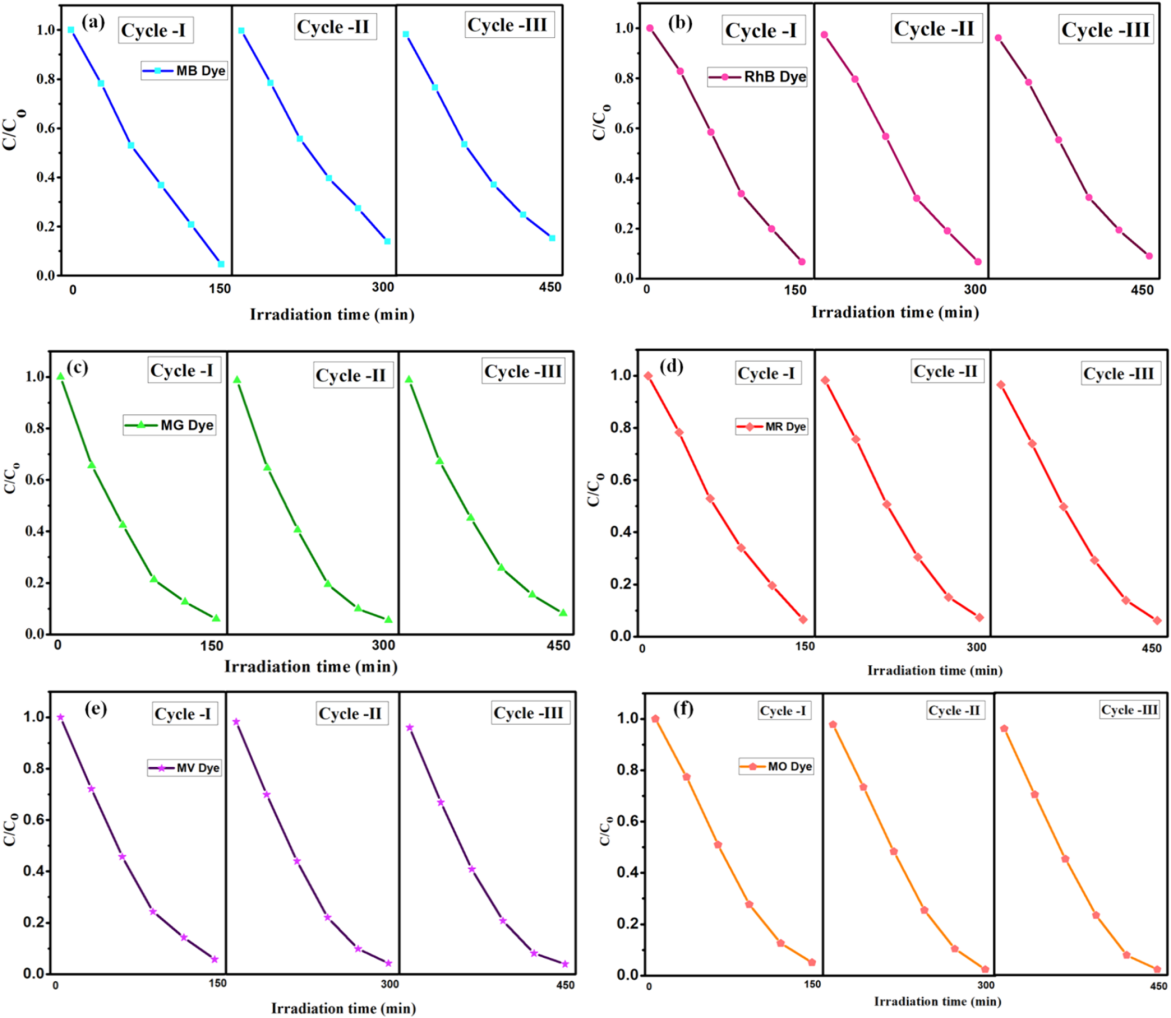
Regeneration study for (a) MB, (b) RhB, (c) MG, (d) MR, (e) MV, and (f) MO dyes for La_2_O_3_ nanoparticles calcinated for 5 h.

### Scavenger Test

Figure 12 shows the radical scavenger tests carried out using La_2_O_3_ nanoparticles calcinated for 5 h; the roles of different reactive oxygen species (ROS) varied depending on the type of dye being degraded. For methylene blue (MB), the degradation efficiency dropped the most when isopropyl alcohol (IPA), a known hydroxyl radical (^•^OH) scavenger, was added. This clearly points to ^•^OH being the main reactive species breaking down MB, with superoxide radicals (^•^O_2_^–^) and photogenerated holes (h^+^) playing smaller, supporting roles.^48^ In the case of rhodamine B (RhB), it was a different story. Here, the strongest suppression came from benzoquinone (BQ), which traps the superoxide radicals. This suggests that ^•^O_2_^–^ plays the leading role in RhB degradation, a finding that makes sense, as RhB is often broken down through a pathway called *N*-deethylation, which is typically driven by superoxide radicals. Hydroxyl radicals and holes still contribute, but to a lesser extent.^49^

**Figure 12:**
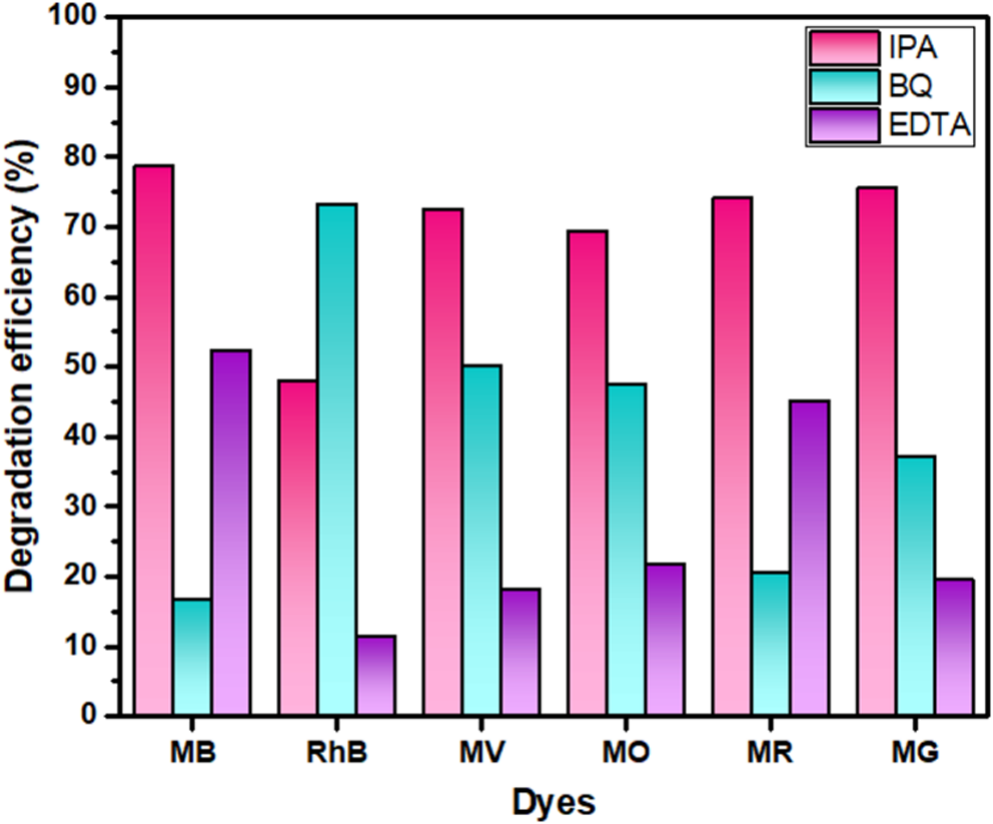
Effect of radical trapping on degradation efficiency.

For methyl violet (MV), the scavenger tests showed that both ^•^OH and ^•^O_2_^–^ were actively involved. When either IPA or BQ was introduced, there was a noticeable decrease in degradation efficiency. This means that MV responds well to attacks from both types of ROS, likely due to its complex triphenylmethane structure, which offers multiple reactive sites. Holes (h^+^), however, had a much smaller effect.^50^ With methyl orange (MO), an anionic azo dye, both hydroxyl and superoxide radicals, again played key roles. The suppression of activity with both IPA and BQ confirmed their involvement. EDTA, which scavenges photogenerated holes, also had a moderate effect, suggesting that holes may assist in the process, especially in breaking the dye’s stable azo bond.^51^ Similarly, methyl red (MR), another azo dye, showed a strong response to IPA, pointing to hydroxyl radicals as primary actors. Superoxide radicals and holes seemed to help along the way, but were not the main players.^52^ Finally, with malachite green (MG), the pattern was similar to that of MB. The addition of IPA led to a significant drop in performance, highlighting the dominant role of ^•^OH radicals in its degradation. BQ had a smaller effect, and EDTA had minimal influence, indicating that superoxide radicals were secondary and holes had very limited involvement.^53^

Overall, these results highlight how the structure and charge of each dye influence which reactive species are most effective during photocatalytic degradation. La_2_O_3_ nanoparticles show strong promise in that they can generate multiple reactive species and adapt to different degradation pathways depending on the pollutant, making them a versatile choice for treating dye-contaminated wastewater.

## Conclusions

This study underscores how a simple adjustment of extending the calcination time can significantly enhance the performance of La_2_O_3_ nanoparticles as photocatalysts for wastewater treatment. By increasing the calcination time from 3 to 5 h, we observed a substantial growth in crystallite size from 32.79 to 45.49 nm, leading to better crystallinity and structural stability. At the same time, the bandgap energy decreased from 4.6 to 4.4 eV, making the nanoparticles more effective in absorbing light and generating reactive species needed for pollutant degradation. The impact of these improvements was evident in photocatalytic tests, where La_2_O_3_ nanoparticles calcinated for 5 h achieved over 95.1% for MB, 93.2% for RhB, 94% for MG, 93.3% for MR, 94.2% for MV, and 94.8% for MO in just 150 min. Kinetic analysis confirmed a fast reaction rate, with rate constants as high as 1.88 × 10^–2^ min^–1^ for MV and 1.89 × 10^–2^ min^–1^ for MO, highlighting their efficiency in breaking down pollutants. Scavenger studies further revealed that hydroxyl radicals (^•^OH) were the most influential in driving the degradation process, followed by superoxide radicals (^•^O_2_^–^) and photogenerated holes (h^+^), offering mechanistic validation of the observed photocatalytic efficiency. Moreover, these nanoparticles demonstrated impressive durability, maintaining over 90% of their photocatalytic efficiency even after three reuse cycles, making them a reliable choice for real-world applications. These findings not only validate La_2_O_3_ nanoparticles as a cost-effective and sustainable alternative to traditional photocatalysts but also open new possibilities for improving water purification technologies. By fine-tuning synthesis conditions and exploring material modifications, La_2_O_3_-based systems could play a pivotal role in addressing industrial wastewater challenges, bringing us closer to a cleaner and greener future.
